# Disparidade de Gênero na Autoria Principal e Sênior em Periódicos Brasileiros de Cardiologia

**DOI:** 10.36660/abc.20220058

**Published:** 2022-11-23

**Authors:** Claudio Tinoco Mesquita, Aline Goneli de Lacerda, Isabella Carolina de Almeida Barros Urel, Eliete Dalla Corte Frantz, Vinícius de Pádua Vieira Alves, Luana Evelyn de Oliveira Amorim, Bruna de Almeida Coutinho, Letícia Rodrigues Dalben, Juliana Cadilho da Silva Abrantes, Vanessa Dias Veloso, Luíza Lucchesi Cabral de Mello, Gláucia Maria Moraes de Oliveira, Fernando de Amorim Fernandes

**Affiliations:** 1 Universidade Federal Fluminense Universitário Antônio Pedro/EBSERH Niterói RJ Brasil Universidade Federal Fluminense - Hospital Universitário Antônio Pedro/EBSERH, Niterói, RJ – Brasil; 2 Universidade Federal Fluminense Niterói RJ Brasil Universidade Federal Fluminense, Niterói, RJ – Brasil; 3 Universidade Federal do Rio de Janeiro Rio de Janeiro RJ Brasil Universidade Federal do Rio de Janeiro, Rio de Janeiro, RJ – Brasil

**Keywords:** Publicações Científicas, Autoria e Co-autoria de Publicações Científicas, Análise de Gênero, Disparidade, Equidade de Gênero

## Abstract

**Fundamento:**

Apesar da importância das mulheres na pesquisa clínica, não existe uma avaliação da fração de mulheres em posições de autoria nos periódicos de cardiologia da SBC.

**Objetivos:**

Avaliar a fração de mulheres autoras na International Journal of Cardiovascular Sciences (IJCS) e nos Arquivos Brasileiros de Cardiologia (ABC Cardiol) nas últimas décadas.

**Métodos:**

Realizamos busca dos artigos originais dos ABC Cardiol, entre 2000 e 2019, e da IJCS, entre 2010 e 2019. Foi feito levantamento do número de primeiras e últimas autoras e do total de artigos originais de 2010 a 2019. Calculamos as proporções totais de autorias femininas e comparamos o primeiro quinquênio com o segundo. Para avaliar a evolução temporal das duas décadas, analisamos apenas dados dos ABC Cardiol. Utilizamos o teste Qui-quadrado para analisar as diferenças dentro de cada revista e entre ambas. O *software* IBM® SPSS® foi utilizado nas análises. O nível de significância adotado foi de 5%.

**Resultados:**

De 2010 a 2019, foram publicados 1157 artigos originais nos ABC Cardiol e 398 na IJCS. Observamos que as mulheres têm maior predominância como primeiras autoras na IJCS em relação aos ABC Cardiol, mas os homens predominam como últimos autores em ambos. De 2010 a 2019, não houve modificação significativa na proporção de autorias femininas. Ao longo das décadas analisadas para os ABC Cardiol, houve projeção de crescimento linear de autorias femininas, sendo que a inclinação da reta é maior na projeção da primeira autoria que na autoria sênior.

**Conclusões:**

Há disparidade de gênero com menor representatividade feminina nas autorias dos artigos dos periódicos cardiológicos brasileiros analisados: Arquivos Brasileiros de Cardiologia e *International Journal of Cardiovascular Sciences*. Acreditamos que a partir destes resultados mais esforços devam ser implementados em busca de equidade de gênero na produção científica cardiológica veiculada por estes periódicos.

## Introdução

As mulheres na medicina acadêmica continuam tendo sub-representação e enfrentam grandes desafios profissionais. Apesar do aumento progressivo na proporção de mulheres graduadas em medicina, elas têm menor probabilidade de ascenderem a cargos de chefia dentro da medicina acadêmica, menos chances de serem reconhecidas como especialistas e líderes, e menores chances de serem convidadas para apresentações em conferências médicas nacionais ou mesmo de receberem prêmios de prestígio.^1,2^ Ouyang et al.,^3^ utilizaram um extenso banco de dados de publicações e concluíram que, apesar de a representação feminina nas pesquisas publicadas na área da cardiologia ter aumentado nas últimas quatro décadas, há uma lacuna persistente na representação das mulheres na pesquisa em todos os níveis, seja como primeira autoria, autoras sêniores e em relação ao número de publicações. Outra observação interessante de Asghar et al.,^4^ foi que as autoras do sexo feminino têm maior probabilidade de ter uma mentora em comparação com seus colegas do sexo masculino. Estes autores concluíram que as posições de liderança feminina provavelmente influenciam de modo positivo outras mulheres em seus departamentos e motivam o envolvimento com a pesquisa científica de modo mais intenso.

Moraes, Kovacs^5^ traçaram um paralelo entre o Brasil e os Estados Unidos destacando que, embora as mulheres representem metade da população, apenas um terço dos cardiologistas são mulheres, mesmo com as doenças cardiovasculares respondendo por cerca de 30% das causas de mortalidade em nosso país e por um terço das mortes de mulheres no mundo. Segundo o relatório da Elsevier intitulado *The Researcher Journey Through a Gender Lens*^6^ (A jornada do pesquisador através de lentes de gênero), atualizado em novembro de 2020, a desigualdade de gênero pode ser observada em termos de resultados de publicações, citações, bolsas concedidas e colaborações. Em todos os países incluídos no estudo, a porcentagem de mulheres que publicaram em periódicos internacionais é menor do que a de homens. Com relação às citações dos artigos, há ainda uma diferença de gênero: trabalhos de autoria de mulheres são citados com menos frequência do que de homens. Quando avaliamos os estudos de graus mais elevados de impacto científico, que são os estudos clínicos randomizados (ECRs), Mehran et al.,^7^ observaram que houve um aumento progressivo no número de mulheres que foram as primeiras autoras de ECRs de cardiologia no período de 2011 a 2020; passando de pouco mais de 20% dos artigos para 30% ao final da década. Os autores creditam este aumento à defesa do empoderamento feminino e da representação igualitária dos gêneros.

Conforme a “Carta das Mulheres”, documento publicado por de Oliveira et al.,^8^ nos Arquivos Brasileiros de Cardiologia, é de suma importância fomentar atividades voltadas para a consolidação da cardiologia entre as mulheres brasileiras, a fim de multiplicar as oportunidades do cuidado na perspectiva feminina, permitindo a integração e a troca de experiências que amplifiquem a melhoria da prática clínica diária. Lançado em 1948, os ABC Cardiol é um dos principais veículos de divulgação das pesquisas científicas brasileiras na área das ciências cardiovasculares. O *International Journal of Cardiovascular Sciences* (IJCS) é um periódico incorporado à Sociedade Brasileira de Cardiologia em 2015, que foi derivado da Revista Brasileira de Cardiologia, criada em 2010 pela Sociedade de Cardiologia do Estado do Rio de Janeiro. Juntos, estes periódicos publicam grande parte da produção científica na área da cardiologia brasileira, em especial os produtos da pós-graduação *stricto sensu*. A despeito da importância substancial das mulheres na prestação de cuidados de saúde e na realização da pesquisa clínica em todo o mundo, não existe uma avaliação da fração de mulheres que ocupam posições de primeira autoria e autoria sênior nos periódicos de cardiologia mantidos pela Sociedade Brasileira de Cardiologia, ABC Cardiol e IJCS. A avaliação deste perfil e da sua variação ao longo das últimas duas décadas pode identificar disparidades autorais entre homens e mulheres em periódicos nacionais na área de cardiologia e pode permitir o desenvolvimento de estratégias para redução das barreiras à representação feminina na liderança médica, bem como na promoção acadêmica na área de cardiologia. O objetivo deste artigo é avaliar o papel das mulheres como autoras de artigos científicos em cardiologia nos periódicos da Sociedade Brasileira de Cardiologia para que estes dados possam ser a base para aumentar a inclusão das mulheres na produção científica cardiológica.

## Métodos

Realizamos um estudo de corte transversal, em que foi realizada busca bibliográfica de todos os artigos originais publicados na revista ABC Cardiol entre os anos 2000 e 2019 e de todos os artigos originais publicados na *IJCS* entre os anos 2010 e 2019 nos sites das revistas mencionadas.^9,10^ A coleta de dados foi realizada entre dezembro de 2020 e fevereiro de 2021 nas bases de dados dos endereços eletrônicos correspondentes de cada revista. O gênero dos(as) autores(as) foi determinado pela inspeção do nome do(a) primeiro(a) autor(a) e do(a) último(a) autor(a) (sênior). Em casos de incerteza do gênero, foi realizada a pesquisa do nome do(a) autor(a) em website da sua respectiva instituição ou em redes sociais.

Em todas as etapas, dois ou mais pesquisadores atuaram de forma independente e as discordâncias foram resolvidas por consenso.

### Critérios de elegibilidade para a seleção dos artigos

Os seguintes critérios foram usados para inclusão de artigos nesta revisão: 1) artigos originais, 2) publicações feitas entre 2000 e 2019 nos ABC Cardiol, 3) publicações feitas entre 2010-2019 na Revista IJCS. Os artigos foram excluídos se fossem editoriais, minieditoriais, revisões ou artigos especiais.

### Extração de dados

Os investigadores, depois da busca e exclusão dos artigos não pertinentes, extraíram independentemente os dados dos estudos selecionados, de acordo com roteiro pré-estabelecido. Foram coletados o número de autoras mulheres dos artigos, o número de autores homens dos artigos, o número e identificação de todas as mulheres primeiras autoras dos artigos, número e identificação de todas as mulheres últimas autoras dos artigos.

### Análise estatística

Foram utilizados dados numéricos para a quantificação dos números absolutos de primeiras e últimas autoras e do total de artigos originais nos periódicos do ano 2010 até 2019 para os artigos dos ABC Cardiol e para os artigos do IJCS. As variáveis categóricas serão apresentadas através de números absolutos e percentagens. A partir dos dados obtidos foram calculadas as proporções totais de autorias femininas de acordo com os periódicos e comparada a evolução temporal dentro da década das autorias comparando o primeiro quinquênio com o segundo. As proporções de autorias femininas e masculinas foram comparadas entre a primeira metade do período com a segunda metade. No caso dos dados dos ABC Cardiol foram analisadas duas décadas (2000 a 2019); enquanto no IJCS foi analisado apenas o período de 2010 a 2019, pois o IJCS foi criado no ano 2010. Para analisar as diferenças de proporções das autorias dentro de cada revista e entre as duas revistas foi utilizado o teste Qui-quadrado. As análises foram realizadas com o *software* IBM® SPSS® versão 21. O nível de significância adotado foi de 5%.

### Aspectos bioéticos

Foram utilizados apenas dados públicos que estão depositados nos websites dos periódicos cardiológicos desta pesquisa, respeitando o item III da Resolução CNS 510/2016 que define que as pesquisas que utilizam dados de domínio público não necessitam de avaliação pelo sistema CEP/CONEP.

## Resultados

A Tabela 1 apresenta o número de artigos originais encontrados no período de 2010 a 2019 para os ABC Cardiol e IJCS de acordo com a autoria e o gênero. Foram publicados no período 1157 artigos originais nos ABC Cardiol e 398 artigos originais no IJCS. Observamos uma predominância de homens como primeiros autores de artigos nos ABC (666 autores homens; 58%) enquanto na IJCS há uma discreta predominância de primeiras autoras mulheres (212 autoras mulheres; 53%). Esta diferença entre os periódicos é estatisticamente significativa (p = 0,001; Tabela 1) indicando que as mulheres têm maior predominância como autoras no IJCS em relação aos ABC Cardiol.

**Tabela 1:** Número e percentual de artigos originais publicados nos Arquivos Brasileiros de Cardiologia e *International Journal of Cardiovascular Sciences* de acordo com o gênero dos autores entre 2010 e 2019

Artigos	Total de artigos originais	Primeira autoria feminina (%)	Primeira autoria masculina (%)	Última autoria feminina (%)	Última autoria masculina (%)
ABC Cardiol	1157	491 (42%)	666 (58%)	284 (25%)	873 (75%) *
IJCS	398	212 (53%)	196 (47%)	163 (41%)	235 (59%) *
TOTAL	1718	771 (45%)	947 (55%)	494 (29%)	1224 (71%)

ABC Cardiol: Arquivos Brasileiros de Cardiologia; IJCS: International Journal of Cardiovascular Sciences.

* comparação entre última autoria masculina ABC x IJCS: p < 0,001.

Ao analisar a autoria sênior dos artigos, observamos que os homens predominam como últimos autores em ambos os periódicos. Entretanto, a frequência de homens como últimos autores nos ABC Cardiol é significativamente maior do que a frequência de homens como últimos autores no IJCS (873 autores nos ABC Cardiol - 75% x 235 autores no IJCS - 59%; valor de p < 0,001; Tabela 1).

A Tabela 2 apresenta a comparação de gênero das autorias do IJCS no período de 2010 a 2019, dividindo a década em dois quinquênios. Observamos que não houve modificação significativa na proporção de autorias femininas tanto na primeira posição do artigo (55% no primeiro quinquênio x 52% no segundo quinquênio; p = 0,2) quanto na última posição (42% no primeiro quinquênio x 40% no segundo quinquênio; p = 0,8).

**Tabela 2:** Número e percentual de artigos originais publicados nos *International Journal of Cardiovascular Sciences* de acordo com o gênero dos autores entre 2010 e 2019 divididos de acordo com os quinquênios da década

Artigos	Total de artigos originais	Primeira autoria feminina (%)	Primeira autoria masculina (%)	Última autoria feminina (%)	Última autoria masculina (%)
IJCS 2010-2014	160	88 (55%)	72 (45%)	67 (42%)	93 (58%)
IJCS 2015-2019	238	124 (52%)	112 (48%)	96 (40%)	142 (60%)

IJCS: International Journal of Cardiovascular Sciences.

A Tabela 3 apresenta a comparação de gênero das autorias dos ABC Cardiol no período de 2010 a 2019, dividindo a década em dois quinquênios. Observamos que não houve modificação significativa na proporção de autorias femininas tanto na primeira posição do artigo (42% no primeiro quinquênio x 42% no segundo quinquênio; p=1) quanto na última posição (25% no primeiro quinquênio x 24% no segundo quinquênio; p = 0,8).

**Tabela 3:** Número e percentual de artigos originais publicados nos Arquivos Brasileiros de Cardiologia de acordo com o gênero dos autores entre 2010 e 2019 divididos de acordo com os quinquênios da década:

Artigos	Total de artigos originais	Primeira autoria feminina (%)	Primeira autoria masculina (%)	Última autoria feminina (%)	Última autoria masculina (%)
ABC Cardiol 2010-2014	656	279 (42%)	377 (58%)	163 (25%)	493 (75%)
ABC Cardiol 2015-2019	501	212 (42%)	289 (58%)	121 (24%)	380 (76%)

ABC Cardiol: Arquivos Brasileiros de Cardiologia.

A Tabela 4 apresenta a comparação de gênero das autorias dos ABC Cardiol ao longo do tempo, considerando a década de 2000 a 2009 com 2010 à 2019. Observamos um aumento significativo na proporção de autorias femininas tanto na primeira (33% na década de 2000 x 42% na década de 2010; p < 0,0001) quanto na última posição (20% na década de 2000 x 25% na década de 2010; p = 0,006).

**Tabela 4:** Número e percentual de artigos originais publicados nos Arquivos Brasileiros de Cardiologia de acordo com o gênero dos autores comparando a década de 2000 à década de 2010

Artigos	Total de artigos originais	Primeira autoria feminina (%)	Primeira autoria masculina (%)	Última autoria feminina (%)	Última autoria masculina (%)
ABC Cardiol 2000-2009	1026	340 (33%)	686 (77%)	202 (20%)	824 (80%)
ABC Cardiol 2010-2019	1157	491 (42%)	666 (58%)	284 (25%)	873 (75%)

ABC Cardiol: Arquivos Brasileiros de Cardiologia.

**Figura 1:**
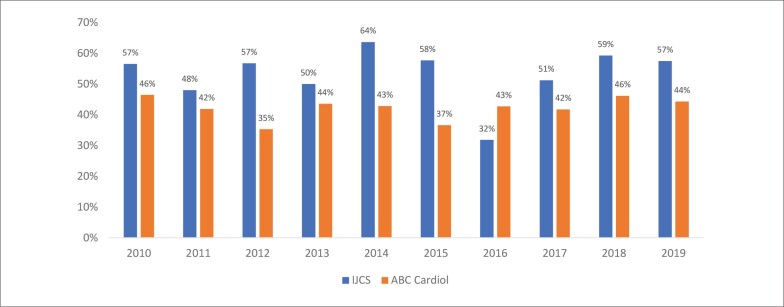
Evolução da proporção de primeiras autorias femininas entre 2010 e 2019 nos periódicos IJCS e ABC Cardiol. ABC Cardiol: Arquivos Brasileiros de Cardiologia; IJCS: International Journal of Cardiovascular Sciences.

As Figuras 1 e 2 demonstram a evolução temporal, ano a ano, das primeiras e últimas autorias femininas, respectivamente, nos periódicos analisados na década de 2010 - 2019. Ambas as Figuras apresentam uma distribuição variável ao longo do período analisado, sem estabelecer um perfil padrão das autorias femininas, independentemente da posição em ambas os periódicos da área de cardiologia.

**Figura 2:**
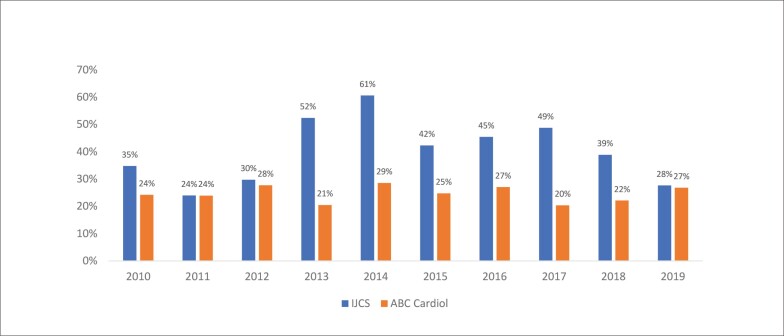
Evolução da proporção de últimas autorias femininas entre 2010 e 2019 nos periódicos IJCS e ABC Cardiol. ABC Cardiol: Arquivos Brasileiros de Cardiologia; IJCS: International Journal of Cardiovascular Sciences.

**Figura 3:**
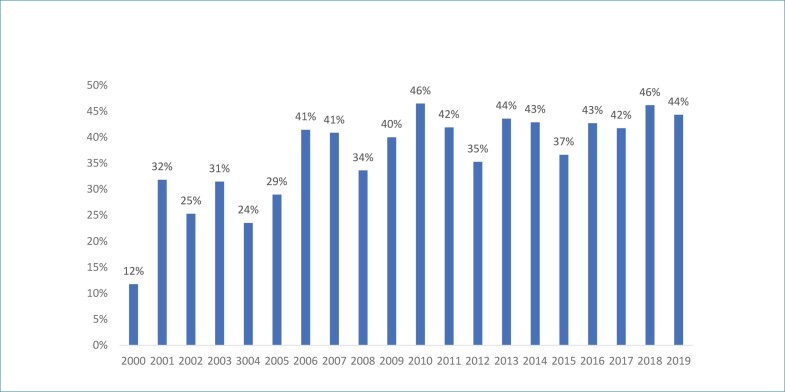
Evolução temporal da proporção da primeira autoria feminina no período de 2000 e 2019, no periódico ABC Cardiol. ABC Cardiol: Arquivos Brasileiros de Cardiologia.

**Figura 4:**
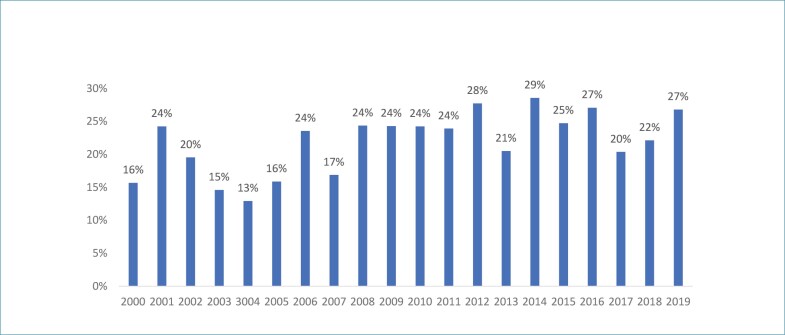
Evolução temporal da proporção da última autoria feminina no período de 2000 e 2019, no periódico ABC Cardiol. ABC Cardiol: Arquivos Brasileiros de Cardiologia.

As Figuras 3 e 4 representam a evolução temporal, ano a ano, da primeira e última autoria, respectivamente, ao longo das duas décadas analisadas para os artigos publicados no periódico ABC Cardiol. Observamos que há uma sazonalidade com relação ao número de autorias femininas tanto na primeira (Figura 3) quanto na última posição (Figura 4) de autores dos artigos originais publicados no período analisado sem configurar uma tendência clara de mudança.

## Discussão

O objetivo do presente estudo foi investigar a diversidade de gênero nas publicações dos principais periódicos das pesquisas brasileiras, na área das ciências cardiovasculares, nas últimas décadas. Nossos achados demostraram uma disparidade de gênero na autoria dos artigos, tanto na primeira (45% de autoras) quanto na última posição (29% de autoras), apontando uma representatividade minoritária feminina. Contudo, mesmo que discreta, nossos resultados sugerem uma crescente participação feminina nas principais posições de autoria durante as últimas décadas, obviamente aquém da equidade de gênero desejada.

O ambiente acadêmico tem presenciado maior número de mulheres cientistas no Brasil nas mais variadas áreas. Haja visto o censo de 2016 do Diretório dos Grupos de Pesquisa no Brasil do Conselho Nacional de Desenvolvimento Científico e Tecnológico (CNPq),^11^ o qual mostra que cerca de 50% do total de pesquisadores é do sexo feminino. Porém, a representatividade feminina diminui à medida que a carreira científica avança, principalmente em posições de liderança, atingindo 45% do total de líderes de grupos de pesquisa brasileiros. Fato corroborado pelo presente estudo que mostrou a sub-representação feminina nas diferentes posições de autoria, alcançando índices mais próximos da equidade de gênero na primeira autoria feminina (45% do total de artigos publicados no ABC Cardiol e IJCS) e uma disparidade mais evidente em posições de liderança, como na autoria sênior (apenas 29% do total de artigos publicados no ABC Cardiol e IJCS) das produções científicas brasileiras, na área das ciências cardiovasculares, nas últimas décadas. Destacando-se ainda que o IJCS apresenta maior representatividade feminina, tanto na primeira quanto na última posição de autoria, em relação ao ABC Cardiol no total de artigos originais publicados na última década. Os dados do nosso estudo se comparam favoravelmente com os apresentados no estudo de Mehran et al.,^7^ que encontraram no ano de 2019 a proporção de 30% dos artigos sobre estudos randomizados em cardiologia como tendo primeira autoria feminina.

Dentre as principais causas da disparidade de gênero no desempenho acadêmico estão o viés implícito e a ameaça pelo estereótipo.^12^ Mulheres e outros grupos étnicos e sociais comumente não se encaixam nas percepções das qualidades de cientistas de sucesso, desencadeando estereótipos culturais negativos, mesmo que sem consciência, de fraco desempenho científico, sem relação com a verdadeira capacidade. O impacto destas atitudes e julgamentos, principalmente em relação ao gênero, acabam implicitamente influenciando ambientes acadêmicos, nos quais os homens geralmente predominam em posições de prestígio.^12,13^ Além disso, o importante trabalho desempenhado pelo Movimento *Parent in Science*^14^ (https://www.parentinscience.com/) destaca a maternidade como um dos fatores mais importantes para a subrepresentatividade das mulheres na ciência, contribuindo com reduções na produtividade de artigos científicos e depósitos de patentes, por exemplo.

Outro fato evidenciado no agravamento da disparidade de gênero no contexto da pandemia de Covid-19 é que as mulheres não têm ocupado papeis de liderança em ensaios clínicos internacionais. Chatterjee, Werner^15^ analisaram 1.548 estudos relacionados à pandemia e concluíram que apenas 27,8% deles foram liderados por mulheres, sendo menos de um terço dos ensaios clínicos sobre Covid-19 realizados por mulheres. Cabe ressaltar ainda que o levantamento realizado no Brasil durante o isolamento social relativo à Covid-19 (abril e maio de 2020) enfatizou que mulheres com filhos tiveram a produtividade acadêmica mais negativamente afetadas pela pandemia.^16^ Diante deste fato, o presente estudo não incluiu o período da pandemia na análise (publicações de 2020 e 2021), acreditando que merece atenção diferenciada e será foco de um estudo futuro do grupo, já em andamento.

Por outro lado, nos últimos anos tem se promovido inúmeras iniciativas na tentativa de desencadear mudanças para amenizar a desigualdade de gênero na ciência brasileira. A exemplo da inclusão do período de licença maternidade no Currículo Lattes, tornando a seleção de pesquisadores com base nesta ferramenta mais inclusiva.^17^ Nesta interface, mesmo que longe do ideal, mostramos uma projeção linear crescente ao longo dos anos na representatividade feminina, principalmente na posição de primeira autoria nas publicações do ABC. Em uma visão otimista, possivelmente com impacto positivo das iniciativas mencionadas acima, podemos projetar uma participação maior das mulheres nos cargos de liderança e nas principais posições de autoria das publicações científicas.

Este levantamento de dados sobre a ordem de autoria por gênero dos principais periódicos das pesquisas brasileiras atualmente, na área das ciências cardiovasculares, revelou a sub-representação feminina na produção científica. Esperamos que o presente estudo estimule reflexões sobre o grande desafio pela busca da equidade de gêneros em uma comunidade mais diversificada e inclusiva na ciência.

Entre as limitações encontradas para a realização do estudo foi que a análise não levou em conta a idade dos autores ou o tempo de formação. Isto pode ser importante tendo em vista que tem havido um aumento progressivo de mulheres médicas. Isto faz com que haja uma proporção maior de homens com mestrado e doutorado em comparação com as mulheres médicas, o que ainda é mais crítico com o fato de que no Brasil estes cursos estão ligados diretamente com a produção científica.^18^ Outra limitação foi a incapacidade de correlacionar a produção científica de modo regional identificando áreas do Brasil em que haja maior disparidade de gênero e que mereçam maior estudo. Entretanto os resultados deste estudo são únicos e pioneiros apontando a necessidade de ações que aumentem a inclusão das mulheres na autoria da produção científica cardiológica.

## Conclusão

Há disparidade de gênero com menor representatividade feminina nas autorias dos artigos dos periódicos cardiológicos brasileiros analisados: Arquivos Brasileiros de Cardiologia e *International Journal of Cardiovascular Sciences*. Acreditamos que a partir destes resultados mais esforços devam ser implementados em busca de equidade de gênero na produção científica cardiológica veiculada por estes periódicos.
