# Supplementary material for: Disparidade de Gênero na Autoria Principal e Sênior em Periódicos Brasileiros de Cardiologia
Source: Arq Bras Cardiol. 2022 Nov 23;119(6):960–7. [Article in Portuguese] doi: 10.36660/abc.20220058 (PMC9814799; doi:10.36660/abc.20220058)
Supplement: Translation

# Translation: Gender Disparity in First and Senior Authorship in Brazilian Cardiology Journals

**Authors:** Claudio Tinoco Mesquita, Aline Goneli de Lacerda, Isabella Carolina de Almeida Barros Urel, Eliete Dalla Corte Frantz, Vinícius de Pádua Vieira Alves, Luana Evelyn de Oliveira Amorim, Bruna de Almeida Coutinho, Letícia Rodrigues Dalben, Juliana Cadilho da Silva Abrantes, Vanessa Dias Veloso, Luíza Lucchesi Cabral de Mello, Gláucia Maria Moraes de Oliveira, Fernando de Amorim Fernandes

**Affiliations:** 1 Niterói RJ Brasil Universidade Federal Fluminense - Hospital Universitário Antônio Pedro/EBSERH, Niterói, RJ – Brazil; 2 Niterói RJ Brasil Universidade Federal Fluminense, Niterói, RJ – Brazil; 3 Rio de Janeiro RJ Brasil Universidade Federal do Rio de Janeiro, Rio de Janeiro, RJ – Brazil

**Keywords:** Scientific Publications, Authorship and Co-Authorship in Scientific Publications, Gender Analysis, Disparity, Gender Equity

## Abstract

**Background:**

Despite the importance of women in clinical research, no assessment has been made of the fraction of women in a leadership positions in the Cardiology journals of the SBC.

**Objectives:**

To assess the fraction of female authors in the International Journal of Cardiovascular Sciences (IJCS) and the *Arquivos Brasileiros de Cardiologia* (ABC Cardiol) over the last decades.

**Methods:**

We searched the original articles of the ABC Cardiol, from 2000 to 2019, and of the IJCS, from 2010 to 2019. We surveyed the number of first and senior female authors and the total number of original articles from 2010 to 2019. We calculated the total proportion of female authorship and compared the first quinquennium with the second. Only data from the ABC Cardiol were analyzed to assess the temporal evolution of the two decades. We used the chi-square test to assess the differences within each journal and between them. The IBM® SPSS® software was used in the analyses. The level of significance adopted was 5%.

**Results:**

From 2010 to 2019, 1,157 original articles were published in the ABC Cardiol and 398 in the IJCS. We observed that women are more prevalent as first authors in the IJCS compared to the ABC Cardiol, but men prevail as senior authors in both journals. From 2010 to 2019, there was no significant change in the proportion of female authorship. Throughout the decades analyzed for the ABC Cardiol, there was a projection of linear growth of female authorship, with the slope of the line being greater in the first authorship than in senior authorship.

**Conclusions:**

There is gender disparity, with lower female representativeness in authorship in the articles from the Brazilian Cardiology journals analyzed: *Arquivos Brasileiros de Cardiologia* and International Journal of Cardiovascular Sciences. We believe that based on these results, more efforts should be implemented in the search for gender equity in the cardiology scientific production published by these journals.

## Introduction

Women in Academic Medicine are still underrepresented and face great professional challenges. Although the progressive growth in the proportion of women who graduated in Medicine, they are less likely to occupy leadership positions in Academic Medicine, have lower chances of being recognized as specialists and leaders, and have lower chances of being invited for presentations in national medical conferences or receiving prestige awards.^1,2^ Ouyang et al.^3^ used an extensive database of publications and concluded that although female representation in the research published in the area of Cardiology has increased in the last four decades, there is a persistent gap in women’s representation in research at all levels, whether as first authorship, senior authors and concerning the number of publications. Another interesting observation made by Asghar et al.^4^ was that female authors are more likely to have a female mentor when compared with their male colleagues. These authors concluded that women in leadership positions might positively influence other women in their departments and motivate a more intense involvement with scientific research.

Moraes, Kovacs^5^ traced a parallel between Brazil and the USA, noting that, although women represent half the population, only one-third of cardiologists are women, even with cardiovascular diseases comprising about 30 percent of the causes of death in Brazil and one-third of deaths among women worldwide. According to the report of Elsevier entitled *The Researcher Journey Through a Gender Lens*^6^, upgraded in November 2020, gender inequity can be observed in terms of publications, citations, scholarships awarded and collaborations. In all countries included in the study, the percentage of women who published in international journals is lower compared to men. There is still a gender difference in article citation: female authorship works are less often cited than those authored by men. When we assess higher scientific impact studies, i.e., randomized clinical trials (RCTs), Mehran et al.^7^ observed a progressive increase in the number of female first authors of cardiology RCTs from 2011 to 2020; going from 20 percent of the articles to 30 percent at the end of the decade. The authors credit this increase to defending female empowerment and equal gender representation.

Oliveira et al.^8^, in their document named “Carta das Mulheres,” published by the *Arquivos Brasileiros de Cardiologia* (*ABC Cardiol*), recognize the importance of promoting practices aimed at the consolidation of Cardiology among Brazilian women to increase the opportunity of healthcare from the female standpoint, allowing for integration and exchange of experiences which improve daily clinical practice. Launched in 1948, the ABC Cardiol is one of the main vehicles for disseminating Brazilian scientific research in cardiovascular sciences. The International Journal of Cardiovascular Sciences (IJCS) was incorporated by the Brazilian Society of Cardiology in 2015 and was preceded by the Revista Brasileira de Cardiologia, created in 2010 by the Society of Cardiology of the State of Rio de Janeiro (SOCERJ). Altogether these journals publish a great part of Brazilian scientific production in cardiology, especially the products of stricto sensu postgraduate programs. Despite the substantial importance of women for healthcare and clinical research worldwide, there is no assessment of the fraction of women in first author and senior author positions in the Cardiology journals kept by the Brazilian Society of Cardiology, namely ABC Cardiol and IJCS. The assessment of this profile and its variation throughout the last two decades may allow for the identification of authorship disparities between men and women in Brazilian journals in the area of cardiology and enable the development of strategies aimed at reducing the barriers to female representativeness in medical leadership, as well as in the academic promotion in the area of cardiology. The purpose of this article is to evaluate the role of women as authors of cardiology scientific articles in the journals of the Brazilian Society of Cardiology so that these data can serve as the basis to increase women’s inclusion in cardiology scientific production.

## Methods

We carried out a cross-sectional study, where a bibliographic search of all original articles published in the *ABC Cardiol* was performed between 2000 and 2019, and of all the original articles of the IJCS, between 2010 and 2019, in the websites of those journals.^9,10^ Data collection was carried out between December 2020 and February 2021 in the database of the websites of each journal. The gender of the authors was determined by the analysis of the first and senior authors’ names. In cases of uncertainty regarding gender, we searched for the author’s name on the respective institution’s website or social media.

In all stages, data were independently assessed by two or more researchers and discrepancies were resolved by consensus.

### Eligibility criteria for the selection of articles

The following inclusion criteria were used for selecting articles in this review: 1) original articles, 2) publications made between 2000 and 2019 for the ABC, and 3) publications made between 2010-2019 in the IJCS. The articles were excluded if they were editorials, mini-editorials, reviews or special articles.

### Data collection

After searching and excluding the irrelevant articles, the researchers independently collected the data from the selected studies according to a predefined script. The number of female authors of the articles, the number of male authors of the articles, the number and identification of all women as first authors of the articles, and the number and identification of all women as senior authors of the articles.

### Statistical analysis

Numerical data were used to determine the absolute number of first and senior female authors and the total number of original journal articles from 2010 to 2019 for the ABC Cardiol and the IJCS. Categorical variables were presented as absolute numbers and percentages. From the results obtained, the total proportions of female authorships were calculated according to the journals and the temporal evolution within the decade of the authorships by comparing the first quinquennium with the second.

The proportions of female and male authorships were compared between the first half of the period and the second half.

In the case of the data from ABC Cardiol, two decades (2000 to 2019) were analyzed, whereas, for the IJCS, only the period from 2010 to 2019 was analyzed because the IJCS was created in 2010. The chi-square test was used to analyze the differences in proportions of authorships inside each journal and between both journals. Analyses were performed with IBM® SPSS® Statistics version 21. The level of significance adopted was 5%.

### Bioethical aspects

Only public data available on the websites of the cardiology journals were used in this research, in compliance with item 3 of CSN Resolution no. 510/2016, which states that research using public domain data does not require evaluation by the CEP/CONEP system.

## Results

Table 1 presents the number of original articles found between 2010 and 2019 for the ABC Cardiol and the IJCS according to authorship and gender. During that period, 1,157 original articles were published in the ABC Cardiol and 398 in the IJCS. We observed a predominance of male first authors in the ABC Cardiol (666 male authors; 58%), whereas, in the IJCS, there is a discrete predominance of female first authors (212 female authors; 53%). This difference between the journals is statistically significant (p = 0.001; Table 1), indicating that the predominance of women as first authors is greater in the IJCS compared to the ABC Cardiol.

**Table 1:** Number and percentage of original articles published in the *Arquivos Brasileiros de Cardiologia* and the International Journal of Cardiovascular Sciences according to author gender, from 2010 to 2019

Articles	Total of original articles	First female authorship (%)	First male authorship (%)	Senior female authorship (%)	Senior male authorship (%)
ABC Cardiol	1,157	491 (42%)	666 (58%)	284 (25%)	873 (75%) *
IJCS	398	212 (53%)	196 (47%)	163 (41%)	235 (59%) *
TOTAL	1,718	771 (45%)	947 (55%)	494 (29%)	1,224 (71%)

ABC Cardiol: Arquivos Brasileiros de Cardiologia; IJC: International Journal of Cardiovascular Sciences.

* comparison between senior male authorship ABC Cardiol vs. IJCS: p < 0,001.

When we analyze senior authorship, we note that men prevail as senior authors in both journals. However, the number of men as senior authors in the ABC Cardiol is significantly higher than that of men as senior authors in the IJCS (873 authors in the ABC Cardiol - 75% vs. 235 authors in the IJCS - 59%; p value < 0.001; Table 1).

Table 2 compares authorship gender in the IJCS from 2010 to 2019 by dividing the decade into two quinquenniums. We observed that the was no significant change in the proportion of female authorship, both in the first position of the article (55% in the first quinquennium vs. 52% in the second; p = 0.2) and in a senior position (42% in the first quinquennium vs. 40% in the second; p = 0.8).

**Table 2:** Number and percentage of original articles published in the International Journal of Cardiovascular Sciences according to authorship gender from 2010 and 2019, divided according to the quinquenniums of the decade

Articles	Total of original articles	First female authorship (%)	First male authorship (%)	Senior female authorship (%)	Last male authorship (%)
IJCS 2010-2014	160	88 (55%)	72 (45%)	67 (42%)	93 (58%)
IJCS 2015-2019	238	124 (52%)	112 (48%)	96 (40%)	142 (60%)

IJCS: International Journal of Cardiovascular Sciences.

Table 3 compares authorship gender in the ABC Cardiol from 2010 to 2019, splitting the decade into two quinquennials. We observed no significant change in the proportion of female authorship both in the first position of the article (42% in the first quinquennium vs. 42% in the second; p=1) and in a senior position (25% in the first quinquennium vs. 24% in the second; p = 0.8).

**Table 3:** Number and percentage of original articles published in the *Arquivos Brasileiros de Cardiologia* according to the gender of the author from 2010 to 2019 divided according to the decade quinquenniums

Articles	Total of original articles	First female authorship (%)	First male authorship (%)	Senior female authorship (%)	Senior male authorship (%)
ABC Cardiol 2010-2014	656	279 (42%)	377 (58%)	163 (25%)	493 (75%)
ABC Cardiol 2015-2019	501	212 (42%)	289 (58%)	121 (24%)	380 (76%)

ABC Cardiol: Arquivos Brasileiros de Cardiologia.

Table 4 compares authorship gender in the ABC Cardiol over time, considering the decade from 2000 to 2009 to 2010 to 2019. We observed a significant increase in the proportion of female authorship both in first (33% in the 2000s vs. 42% in the 2010s; p < 0.0001) and in a senior position (20% in the 2000s vs. 25% in the 2010s; p = 0.006).

**Table 4:** Number and percentage of original articles published in the *Arquivos Brasileiros de Cardiologia* according to authorship gender comparing the 2000s with the 2010s

Articles	Total of original articles	First female authorship (%)	First male authorship (%)	Senior female authorship (%)	Senior male authorship (%)
ABC Cardiol 2000-2009	1,026	340 (33%)	686 (77%)	202 (20%)	824 (80%)
ABC Cardiol 2010-2019	1,157	491 (42%)	666 (58%)	284 (25%)	873 (75%)

ABC Cardiol: Arquivos Brasileiros de Cardiologia.

Figures 1 and 2 show the temporal evolution, year by year, of first and senior female authorships, respectively, in the journals analyzed from 2010 to 2019. Both Figures show a variable distribution throughout the period analyzed, without establishing a standard profile of female authorships, regardless of the position in both cardiology journals.

**Figure 1:** Evolution of the proportion of the first female between 2010 and 2019 in the IJCS and ABC Cardiol. ABC Cardiol: Arquivos Brasileiros de Cardiologia; IJCS: International Journal of Cardiovascular Sciences.

**Figure 2:** Evolution in the proportion of senior female authorship between 2010 and 2019 in the IJCS and ABC Cardiol. ABC Cardiol: Arquivos Brasileiros de Cardiologia; IJCS: International Journal of Cardiovascular Sciences.

Figures 3 and 4 represent the temporal evolution, year by year, of first and senior authorship, respectively, throughout the two decades analyzed for the articles published in the ABC Cardiol journal. We observe a seasonality concerning the number of female authorships in the first (Figure 3) and in the senior position (Figure 4) of authors of original articles published in the analyzed period without configuring a clear change trend.

**Figure 3:** Temporal evolution of the proportion of first female authorship in the period 2000 and 2019, in the ABC Cardiol journal. ABC Cardiol: Arquivos Brasileiros de Cardiologia.

**Figure 4:** Evolution in the proportion of senior female authorship between 2000 and 2019 in the ABC Cardiol. ABC Cardiol: Arquivos Brasileiros de Cardiologia.

## Discussion

This study aimed to investigate gender diversity in the publications of the main journals for Brazilian research in cardiovascular sciences in the last decades. Our findings showed gender disparity in article authorship, in first (45% of female authors) and senior positions (29% of female authors), indicating a minor female representation. However, our results suggest a discrete increase in female participation in main authorship positions during the last decades, which is obviously below the desired gender equality.

The academic environment has witnessed a greater number of female scientists in Brazil in various fields, as demonstrated by the 2016 census of the Directory of Research Groups of the Brazilian National Council for Scientific and Technological Development (CNPq),^11^ which shows that about 50% of the researchers are female. Nevertheless, female representation decreases as they advance in a scientific career, especially in leadership positions, reaching 45% of the total Brazilian research group leaders. This study corroborates this fact, as it showed female underrepresentation in the different authorship positions, reaching levels that are closer to gender equity in first female authorship (45% of the total articles published by the ABC Cardiol and the IJCS) and more evident disparity in leadership positions, as in senior authorship (only 29% of the total articles published by the ABC Cardiol and the IJCS) of Brazilian scientific production, in the field of cardiovascular sciences, over the last decades. We also highlight that the IJCS presents greater female representation, both in first and senior authorship, compared with the ABC Cardiol, considering the total number of original articles published in the last decade. Our data corroborate those found by Mehran et al.,^7^ who showed in 2019 that women authored 30% of the articles on randomized trials in cardiology. Among the main causes of gender disparity in academic performance are implicit biases and stereotype threats.^12^ Women and other ethnic and social groups usually do not fit the perceptions of the qualities of successful scientists, triggering negative cultural stereotypes, even unintentionally, of weak scientific performance, which has no relation with true capacity. The impact of these attitudes and judgments, especially concerning gender, ends up implicitly influencing the academic environment, where men usually predominate in prestigious positions.^12,13^ In addition, the important work developed by the Parent in Science movement^14^ (https://www.parentinscience.com/) indicates that motherhood is one of the main factors for the underrepresentation of women in science, accounting for a decrease in the production of scientific articles and deposit of patents, for instance. Another factor that exacerbates gender disparity in the COVID-19 pandemic context is that women have not occupied leadership roles in international clinical trials. Chatterjee, Werner^15^ analyzed 1,548 related to the pandemic and concluded that only 27.8% of them were led by women, corresponding to less than one-third of the clinical trials on COVID-19 carried out by women. It is also important to point out that the survey done in Brazil during social isolation related to COVID-19 (April and May 2020) showed that women with children had their academic productivity more negatively affected by the pandemic.^16^ Thus, this study did not include the pandemic period in the analysis (publications from 2020 to 2021) because we believe that it deserves differentiated attention and will be the focus of a future study of the group, which is already in progress.

On the other hand, over the last years, several initiatives have been promoted aimed at making changes that minimize gender disparity in Brazilian science. One example is the inclusion of the period corresponding to maternity leave in the Brazilian national resumé database, “Curriculo Lattes,” which makes the selection of researchers based on this tool more inclusive.^17^ In this interface, although far from ideal, we showed a growing linear projection in female representation over the years, especially in first-position authorship in the publications of ABC Cardiol. From an optimistic perspective, possibly with the positive impact of the initiatives abovementioned, we can project greater participation of women in leadership positions and the main authorship positions of scientific publications.

This data survey on the order of authorship by gender in the main Brazilian research journals today in the cardiovascular sciences field showed an underrepresentation of women in scientific production. We hope this study stimulates reflections upon the big challenge in the search for gender equity for a more diverse community and a more inclusive science.

Among the limitations found in carrying out this study was that the study did not consider

the authors’ ages and or graduation years. These data may be important since there has been a progressive increase in the number of female doctors. This could cause a greater proportion of men with master and doctorate degrees compared with female medical doctors, which is still more critical because, in Brazil, these courses have a direct connection with scientific production.^18^ Another limitation is that it was impossible to correlate the scientific production at a regional level, identifying the areas in Brazil where gender disparity is greater and should be studied more. However, the results from this study are the only first to point out the necessity of actions that increase women’s inclusion in cardiology scientific production authorship.

## Conclusion

There is gender disparity, with lower female representativeness in authorship in the articles from the Brazilian Cardiology journals analyzed: *Arquivos Brasileiros de Cardiologia* and International Journal of Cardiovascular Sciences. We believe that from these results, more effort should be put into the search for gender equity in the scientific production on Cardiology published by these journals.
